# Supplementary material for: Reliability and Validity of Non-Instrumental Clinical Assessments for Adults with Oropharyngeal Dysphagia: A Systematic Review
Source: J Clin Med. 2023 Jan 16;12(2):721. doi: 10.3390/jcm12020721 (PMC9861493; doi:10.3390/jcm12020721)
Supplement: Supplementary file 1 [file jcm-12-00721-s001.zip › Supplementary FileS3 COSMIN Definitions.pdf]

Supplementary File S3. Reliability and Validity of Non-instrumental Clinical Assessments for Adults with Oropharyngeal Dysphagia: A systematic review.

Definitions of the nine measurement properties according to COSMIN [1, 2]

DOMAIN	MEASUREMENT PROPERTY
RELIABILITY	***Degree to which the measurement is free from measurement error***
	1) **Internal consistency** Degree of the interrelatedness among the items<br>2) **Reliability** Proportion of the total variance in the measurements which is because of "true" differences between patients<br>3) **Measurement error** Systematic and random error of a patient's score that is not attributed to true changes in the construct to be measured.
VALIDITY	***Degree to which an instrument measures the construct(s) it purports to measure***
	4) **Content validity**<br>Degree to which the content of an instrument is an adequate reflection of the construct to be measured<br>5) **Structural validity**<br>Degree to which the scores of an instrument are an adequate reflection of the dimensionality of the construct to be measured<br>6) **Hypotheses testing**<br>Idem construct validity<br>7) **Cross-cultural validity**<br>Degree to which the performance of the items on a translated or culturally adapted instrument are an adequate reflection of the performance of the items of the original version of the instrument<br>8) **Criterion validity**<br>Degree to which the scores of an instrument are an adequate reflection of a 'gold standard'
RESPONSIVENESS	***Ability of an instrument to detect change over time in the construct to be measured***
	9) **Responsiveness** Idem Responsiveness
Interpretability[a]	*Degree to which one can assign qualitative meaning to an instrument's quantitative scores or change in scores*

[a] Interpretability is not considered a psychometric property.

References

1. Mokkink, L.B., et al., *COSMIN methodology for systematic reviews of patient-reported outcome measures (PROMs).* User manual, 2018. **78**(1).
2. Mokkink, L.B., et al., *The COSMIN study reached international consensus on taxonomy, terminology, and definitions of measurement properties for health-related patient-reported outcomes.* Journal of clinical epidemiology, 2010. **63**(7): p. 737-745.
