# Supplementary material for: Reliability and Validity of Non-Instrumental Clinical Assessments for Adults with Oropharyngeal Dysphagia: A Systematic Review
Source: J Clin Med. 2023 Jan 16;12(2):721. doi: 10.3390/jcm12020721 (PMC9861493; doi:10.3390/jcm12020721)
Supplement: Supplementary file 1 [file jcm-12-00721-s001.zip › Supplementary FileS4 Excluded Measures.pdf]

Supplementary File S4. Reliability and Validity of Non-instrumental Clinical Assessments for Adults with Oropharyngeal Dysphagia: A systematic review.

Overview of excluded measures for OD in adults (*n* = 125)

Measure	Acronym	Exclusion criteria
8-item Brief Bedside Dysphagia Screening Test-Revised [1]	BBDST-R	Screen
Achalasia Quality of Life [2]	ASQ	Measure referred to oesophageal problems
Acute Stroke Dysphagia Screen [3]		Screen
Barnes-Jewish Hospital Stroke Dysphagia Screen [4]	BJH-SDS	Screen
Bedside dysphagia Screen [5]	BDS	Screen
Bedside Screening Tool for Dysphagia [6]	BSTD	Screen
Bedside swallowing assessment [7]		Screen
Bedside swallowing test [8]		Not a clinical measure
Bedside Tests [9]		Screen
Boston Residue and Clearance Scale [10]	BRACS	Instrumental assessment
Brief Bedside Dysphagia Screening Test [11]		Measure not developed in English
Brief Bedside Dysphagia Screening Test-Revised [12]		Screen
Burke Dysphagia Screening Test [13]	BDST	Screen
Caregiver Analysis of Reported Experiences with Swallowing Disorders [14]	CARES	Self-report
Center for Neurologic Study Bulbar Function Scale-Short Form [15]	CNS-BFS	Self-report
Cervical auscultation [16]	CA	Instrumental assessment
Chin-down posture [17]		Screen
Choking Risk Assessment [18]	CRA	Not a clinical measure
Clinical swallow examination [19]	CSE	Not a clinical measure
Deglutition Handicap Index [20]	DHI	Self-report
DePaul Hospital Swallow Screener [21]	DHSS	Screen
Dynamic Index for Grading for Swallow Toxicity [22]	DIGEST	Instrumental assessment
Dysphagia Handicap Index [23]	DHI	Self-report
Dysphagia in Multiple Sclerosis [24]	DYMUS	Self-report

Measure	Acronym	Exclusion criteria
Dysphagia Outcome and Severity Scale [25]	DOSS	Instrumental assessment
Dysphagia Screening tool [26]		Screen
Dysphagia Short Questionnaire [27]	DSQ	Self-report
Dysphagia Stress Test [28]	DST	Measure referred to oesophageal problems
Dysphagia Symptom Questionnaire [29]	DSQ	Self-report
Dysphagia Trained Nurse assessment [30]	DTNAx	Screen
Dysphagia, odynophagia, and voice disabilities tool [31]	DOV	Self-report
Dysphagia-Specific SWAL-QoL [32]	DSWAL-QoL	Self-report
Eating Assessment Tool [33]	EAT-10	Self-report
Eckardt symptom score [34]	ESS	Measure referred to oesophageal problems
European Organisation for Research and Treatment of Cancer Quality of Life Questionnaire [35]	EORTC QLQ C-30	Self-report
Eosinophilic Esophagitis Activity Index [36]	EEsAI	Measure referred to oesophageal problems
Esophageal conduit questionnaire [37]		Self-report
Esophageal Hypersensitivity Scale [38]	EHS	Measure referred to oesophageal problems
Esophageal Symptom Questionnaire[39]	ESQ	Measure referred to oesophageal problems
Evolución de la Disfagia Murciego Rubio [40]	EDMUR	Measure not developed in English
Exeter Dysphagia Assessment Technique [41]		Instrumental assessment
Facial-Oral Tract Therapy [42]	FOTT	Measure not developed in English
Functional Assessment of Cancer Therapy Head & Neck Cancer Symptom Index - 10 Item Version [43]	FHNSI-10	Self-report
Functional Assessment of Cancer Therapy [44]	FACT	Self-report
Functional Independence Measure [45]	FIM	Screen
Functional Outcome Questionnaire for Aphasia [46]	FOQ-A	Not focussed on dysphagia
Glasgow Edinburgh Throat Scale [47]	GETS	Not focussed on dysphagia
Groppo-Lawless nurse-initiated Screen [48]	GDS	Screen
Gugging Swallowing Screen [49]	GuSS	Screen

Measure	Acronym	Exclusion criteria
Head & Neck Survey [50]	H&NS	Self-report
Head and Neck Patient Symptom Checklist [51]	HNSC	Self-report
Head and Neck Cancer Survivors' Assessment of Mealtimes [52]	HN-SAM	Self-report
The Hospital for Special Surgery Dysphagia and Dysphonia Inventory [53]	HSS-DDI	Self-report
Hospital for Special Surgery Speech and Swallow Inventory [54]	HSS-SSI	Self-report
Huntington's Disease Dysphagia Scale [55]	HDDS	Self-report
International Classification of Functioning framework [56]	ICF	Not focussed on dysphagia
Jaw-opening force test [57]	JOFT	Instrumental assessment
Kuchi-Kara Taberu Index [58]	KT Index	Measure not developed in English
Lip Force Meter [59]		Instrumental assessment
M. D. Anderson Symptom Inventory with gastrointestinal (GI) cancer [60]	MDASI-GI	Self-report
M. D. Anderson Symptom Inventory-Head and Neck Module [61]	MDASI-HN	Self-report
Mann Assessment of Swallowing Ability – Oral Cancer [62]	MASA-OC	Measure not developed in English
Maximal phonation time [63]	MPT	Screen
Maxwell Swallow Screen [64]	MSS	Screen
Mayo Dysphagia Questionnaire-30 Day [65]	MDQ-30	Self-report
MD Anderson Dysphagia Inventory [66]	MDADI	Self-report
Mealtime Assessment Scale [67]	MAS	Measure not developed in English
Mealtime Assessment Tool [68]	MAT	Measure not developed in English
Medicines Acceptability Questionnaire [69]	MAQ	Self-report
Minimal Eating Form [70]	MEOF	Screen
Minimal Eating Observation Form – II [71]	MEOF-II	Screen
Minimal Eating Observation and Nutrition Form - Version II [72]	MEONF-II	Screen
Modified 30 ml water-swallowing test [73]		Screen

Measure	Acronym	Exclusion criteria
Medication-related Osteonecrosis of the Jaw Quality of Life Questionnaire [74]	MRONJ-QoL	Not focussed on dysphagia
Munich Swallowing Score [75]	MUCSS	Measure not developed in English
Neuromuscular Disease Swallowing Status Scale [76]	NdSSS	Measure not developed in English
Nurse Dysphagia Screen [77]		Screen
Nurse-administered Screening tool [78]		Screen
Nursing Bedside Dysphagia Screen [79]		Screen
Nursing Bedside Swallowing Screen [80]		Screen
Nursing dysphagia Screening tool and the National Institutes of Health Stroke Scale [81]		Screen
Ohkuma questionnaire [82]		Measure not developed in English
OM Daily Questionnaire [83]	OMDQ	Not focussed on dysphagia
Oropharyngeal Dysphagia Screening questionnaire [84]		Screen
Pharyngeal swallow response time [85]	PSRT	Not focussed on dysphagia
Pneumonia Risk Assessment [86]	PRA	Not a clinical measure
Porch Index of Communicative Ability [87]		Not focussed on dysphagia
Postextubation Dysphagia Screening Tool [88]		Screen
Predictive dysphagia score [89]	PreDyScore	Not a clinical measure
Radboud Oral Motor Inventory for Parkinson's Disease [90]	ROMP	Self-report
Rastreamento de Alteracoes da Degluticao em Idosos [91]	RADI	Measure not developed in English
Rectangle tongue template [92]		Not focussed on dysphagia
Risk of Choking scale [93]	ROC	Not focussed on dysphagia
Royal Adelaide Prognostic Index for Dysphagic Stroke [94]	RAPIDS	Self-report
Royal Brisbane Women's Hospital Dysphagia Screening Tool [95]	RBWH	Screen
Royal Free Hospital Gastrostomy Risk Score [96]	RFHG-RS	Screen
Seniors in the Community: Risk Evaluation for Eating and Nutrition [97]	SCREEN-II	Screen

Measure	Acronym	Exclusion criteria
Signs of Weakness or Asymmetry of Lingual/ facial/ palatal Location, of Wakefulness, and 3 ounce water Drink test [98]	SWALLOW-3D	Screen
Simple swallowing provocation test [99]	SSPT	Screen
Standardized Swallowing Assessment [100]	SSA	Screen
Swallow Battery [101]	SwaB	Screen
Swallow frequency analysis [102]	SFA	Not a clinical measure
Swallowing Clinical Assessment Score in Parkinson's Disease [103]	SCAS-PD	Measure not developed in English
Swallowing Disturbance Questionnaire [104]	SDQ	Self-report
Swallowing Outcomes After Laryngectomy [105]	SOAL	Self-report
Swallowing Quality of Life questionnaire [106]	SWAL-QOL	Self-report
Swedish Watson Dysphagia Scale [107]	S-WDS	Measure not developed in English
The Bazaz scale [108]		Self-report
The Clinical Assessment of Dysphagia in Neurodegeneration [109]	CADN	Screen
The Food Intake LEVEL Scale [110]	FILS	Measure not developed in English
The Functional Communication Measures [111]	FCMs	Not focussed on dysphagia
The Progressive Supranuclear Palsy Clinical Deficits Scale [112]	PSP-CDS	Not focussed on dysphagia
The Scleroderma Logopedic Scale [113]	SLS	Measure not developed in English
The Short Nutritional Assessment Questionnaire [114]	SNAQ	Screen
The Signaleringslijst Verslikken [115]	SV	Measure not developed in English
The water-swallowing test [116]	WST	Screen
The Watson scale [117]		Not focussed on dysphagia
Tongue pressure measurement [118]		Not focussed on dysphagia
Toronto bedside swallowing Screening test [119]	TOR-BSST	Screen
Total Dysphagia Risk Score [120]	TDRS	Screen
Two-Step Thickened Water Test [121]	TTWT	Screen
Unified Parkinsons' Disease Rating Scale [122]	UPDRS	Self-report
Vanderbilt Head and Neck Symptom Survey [123]	VHNSS	Self-report

Measure	Acronym	Exclusion criteria
Voice Handicap Index [124]	VHI	Self-report
Volume Viscosity Swallow Test [125]	V-VST	Screen

References

1. Mandysová, P., H. Trundová, and E. Ehler, *Inter-rater agreement of the brief bedside dysphagia screening test-revised in patients with stroke.* Central European Journal of Nursing & Midwifery, 2016. **7**(2): p. 412-419.
2. Slone, S., et al., *Accuracy of Achalasia Quality of Life and Eckardt scores for assessment of clinical improvement post treatment for achalasia.* Diseases of the Esophagus, 2021. **34**(2).
3. Edmiaston, J., et al., *Validation of a dysphagia screening tool in acute stroke patients.* American journal of Critical Care, 2010. **19**(4): p. 357-64.
4. Edmiaston, J., et al., *A simple bedside stroke dysphagia screen, validated against videofluoroscopy, detects dysphagia and aspiration with high sensitivity.* Journal of Stroke & Cerebrovascular Diseases, 2014. **23**(4): p. 712-716.
5. Posillico, S.E., et al., *Bedside dysphagia screens in patients with traumatic cervical injuries: An ideal tool for an under-recognized problem.* The Journal of Trauma and Acute Care Surgery, 2018. **85**(4): p. 697-703.
6. Immovilli, P., et al., *Diagnostic Accuracy of a Bedside Screening Tool for Dysphagia (BSTD) in Acute Stroke Patients.* Journal of Stroke and Cerebrovascular Diseases, 2021. **30(2) (no pagination)**.
7. Smithard, D.G., et al., *Can bedside assessment reliably exclude aspiration following acute stroke?* Age & Ageing, 1998. **27**(2): p. 99-106.
8. Al Hawat, A., et al., *Validity of cervical auscultation in the screening for aspiration.* Revue de Laryngologie Otologie Rhinologie, 2014. **135(2)**: p. 51-56.
9. Kolb, G. and M. Broker, *State of the art in aspiration assessment and the idea of a new non invasive predictive test for the risk of aspiration in stroke.* Journal of Nutrition, Health and Aging, 2009. **13(5)**: p. 429-433.
10. Kaneoka, A.S., et al., *The Boston Residue and Clearance Scale: Preliminary Reliability and Validity Testing.* Folia Phoniatrica et Logopaedica, 2013. **65**(6): p. 312-317.
11. Mandysova, P., et al., *Development of the Brief Bedside Dysphagia Screening Test in the Czech Republic.* Nursing & Health Sciences, 2011. **13**(4): p. 388-395.
12. Mandysova, P., et al., *Development of the Brief Bedside Dysphagia Screening Test - Revised: a Cross-Sectional Czech Study.* Acta medica (Hradec Kralove) / Universitas Carolina, Facultas Medica Hradec Kralove, 2015. **58(2)**: p. 49-55.
13. DePippo, K.L., M.A. Holas, and M.J. Reding, *The Burke Dysphagia Screening Test: validation of its use in patients with stroke.* Archives of Physical Medicine & Rehabilitation, 1994. **75**(12): p. 1284-1286.
14. Shune, S.E., et al., *Creation and Initial Validation of the Caregiver Analysis of Reported Experiences with Swallowing Disorders (CARES) Screening Tool.* American journal of speech-language pathology, 2020. **29(4)**: p. 2131-2144.
15. Macklin, E.A., et al., *A short-form version of the CNS-BFS, a patient reported outcome for assessing bulbar function in amyotrophic lateral sclerosis.* Amyotrophic Lateral Sclerosis and Frontotemporal Degeneration, 2018. **19(Supplement 1)**: p. 297-298.
16. Bergström, L., P. Svensson, and L. Hartelius, *Cervical auscultation as an adjunct to the clinical swallow examination: a comparison with fibre-optic endoscopic evaluation of swallowing.* International Journal of Speech-Language Pathology, 2014. **16**(5): p. 517-28.
17. Baylow, H.E., et al., *Accuracy of clinical judgment of the chin-down posture for dysphagia during the clinical/bedside assessment as corroborated by videofluoroscopy in adults with acute stroke.* Dysphagia, 2009. **24(4)**: p. 423-433.
18. Sheppard, J.J., et al., *Validation of the Choking Risk Assessment and Pneumonia Risk Assessment for adults with Intellectual and Developmental Disability (IDD).* Research in Developmental Disabilities, 2017. **69**: p. 61-76.
19. Garand, K.L.F., et al., *Assessment Across the Life Span: The Clinical Swallow Evaluation.* American Journal of Speech-Language Pathology, 2020. **S. 29(2)**: p. 919-933.

20. Crestani, S., M. Moerman, and V. Woisard, *The "deglutition handicap index" a self-adminitrated dysphagia-specific quality of life questionnaire: Sensibility to change 1.* Revue de Laryngologie Otologie Rhinologie, 2011. **132(1)**: p. 3-7.
21. Behera, A., et al., *A Validated Swallow Screener for Dysphagia and Aspiration in Patients with Stroke.* Journal of Stroke and Cerebrovascular Diseases, 2018. **27(7)**: p. 1897-1904.
22. Green, C., et al., *Use of novel dysphagia index for grading for swallow toxicity (DIGEST) to assess short term functional swallow outcomes in patients with oropharyngeal carcinoma treated with transoral robotic surgery (TORS).* Dysphagia, 2019. **34(6)**: p. 976.
23. Kim, T.Y., et al., *Clinical Usefulness of the Korean Version of the Dysphagia Handicap Index: Reliability, Validity, and Role as a Screening Test.* Dysphagia., 2021.
24. Bergamaschi, R., et al., *The DYMUS questionnaire for the assessment of dysphagia in multiple sclerosis.* Journal of the Neurological Sciences, 2008. **269(1-2)**: p. 49-53.
25. O'Neil, K.H., et al., *The dysphagia outcome and severity scale.* Dysphagia, 1999. **14(3)**: p. 139-145.
26. Cichero, J.A.Y., S. Heaton, and L. Bassett, *Triaging dysphagia: Nurse screening for dysphagia in an acute hospital. [References]*. 2009: Journal of Clinical Nursing. Vol.18(11), 2009, pp. 1649-1659.
27. Skeppholm, M., et al., *The dysphagia short questionnaire: An instrument for evaluation of dysphagia: A validation study with 12 months' follow-up after anterior cervical spine surgery.* Spine, 2012. **37(11)**: p. 996-1002.
28. Taft, T.H., et al., *The dysphagia stress test for rapid assessment of swallowing difficulties in esophageal conditions.* Neurogastroenterology and Motility, 2019. **31(3) (no pagination)**.
29. Kamat, S., et al., *Qualitative Patient Interviews to Assess Content Validity of the Modified Dysphagia Symptom Questionnaire.* Gastroenterology, 2020. **158(6 Supplement 1)**: p. S-831.
30. Benfield, J.K., et al., *Validation and reliability testing of a dysphagia trained nurse assessment in acute stroke.* Dysphagia, 2019. **34(5)**: p. 764.
31. Sclafani, J.A., et al., *Validity and reliability of a novel patient reported outcome tool to evaluate post-operative dysphagia, odynophagia, and voice (DOV) disability after anterior cervical procedures.* International Journal of Spine Surgery, 2017. **11(5)**: p. 285-292.
32. Simpelaere, I., et al., *Application of the Rasch measurement model on the Dutch version of the swallowing quality-of-life questionnaire (DSWAL-QoL).* Quality of Life Research, 2016. **25(1 Supplement 1)**: p. 54.
33. Nogueira, D., et al., *Measuring Outcomes for Dysphagia: Validity and Reliability of the European Portuguese Eating Assessment Tool (P-EAT-10).* Dysphagia (0179051X), 2015. **30**(5): p. 511-520.
34. Cisternas, D., et al., *Fair reliability of eckardt scores in achalasia and non-achalasia patients: Psychometric properties of the eckardt spanish version in a multicentric study.* Neurogastroenterology and Motility, 2020. **32(6) (no pagination)**.
35. Brunelli, C., et al., *Evaluation of quality of life in patients with malignant dysphagia.* Tumori, 2000. **86(2)**: p. 134-138.
36. Schoepfer, A., et al., *Development of a Symptom-based Activity Index for Eosinophilic Esophagitis: The EEsAI PRO Instrument.* Swiss Medical Weekly, 2014. **206)**: p. 10S.
37. Lee, M.K., et al., *Patient-reported outcome domains for the esophageal CONDUIT report card: A prospective trial to establish domains.* Health and Quality of Life Outcomes, 2018. **16**(1): p. 1-13.
38. Taft, T., et al., *The Esophageal Hypersensitivity Scale (Ehs) Is a Reliable and Valid Patient-Reported Assessment Tool.* Gastroenterology, 2018. **154(6 Supplement 1)**: p. S-735.
39. Kwiatek, M.A., et al., *Predictive validity of the esophageal symptom questionnaire (ESQ) for the absence of patient-reported remission in adult eosinophilic esophagitis: Preliminary findings.* Gastroenterology, 2011. **1)**: p. S242.
40. Murciego, P., P. Giménez Barriga, and N. García Atarés, *[Validation of the EDMUR scale, a tool to quantify the evolution of neurogenic dysphagia].* Nutrición Hospitalaria, 2022.
41. Pinnington, L.L., et al., *Interrater and intrarrater reliability of the Exeter Dysphagia Assessment Technique applied to healthy elderly adults.* Dysphagia, 2000. **15(1)**: p. 6-9.
42. Mortensen, J., D. Jensen, and A. Kjaersgaard, *A validation study of the facial-oral tract therapy swallowing assessment of saliva.* Dysphagia, 2016. **31(2)**: p. 271-272.
43. Shaunfield, S., et al., *Optimizing brief, focused symptom assessment in recurrent or metastatic squamous cell carcinoma of the head and neck (SCCHN): the FHNSI-10.* Journal of Clinical Oncology. Conference, 2018. **36**(15 Supplement 1).

44. Shaunfield, S., et al., *Optimizing brief, focused assessment of priority symptoms and concerns in recurrent and/or metastatic squamous cell carcinoma of the head and neck: Content validation of the Functional Assessment of Cancer Therapy/National Comprehensive Cancer Network Head and Neck Symptom Index-10 (FHNSI-10).* Health Science Reports, 2021. **4(4) (no pagination)**.
45. Jeyaseelan, R.D., M.M. Vargo, and J. Chae, *National Institutes of Health Stroke Scale (NIHSS) as An Early Predictor of Poststroke Dysphagia.* American Journal of Physical Medicine & Rehabilitation, 2015. **7**(6): p. 593-8.
46. Glueckauf, R.L., et al., *Functional Outcome Questionnaire for Aphasia: overview and preliminary psychometric evaluation.* NeuroRehabilitation, 2003. **18**(4): p. 281-290.
47. Takahashi, N., et al., *Reliability and validity of the Japanese version of the Glasgow Edinburgh Throat Scale (GETS-J): Use for a symptom scale of globus sensation.* Auris Nasus Larynx, 2018. **45(5)**: p. 1041-1046.
48. Groppo-Lawless, S., C.C. Davies, and A. Lengerich, *Developing and Testing the Diagnostic Accuracy of a Brief Nursing Dysphagia Screen.* Rehabilitation nursing : the official journal of the Association of Rehabilitation Nurses, 2020. **45(6)**: p. 367-373.
49. Cornwell, P.L., B. Cowie, and R. Geraghty, *Examining nurse-led dysphagia screening tools in the general medical hospital population. [References]*. 2017: Speech, Language and Hearing. Vol.20(1), 2017, pp. 29-37.
50. Gliklich, R.E., T.A. Goldsmith, and G.F. Funk, *Are head and neck specific quality of life measures necessary?* Head Neck, 1997. **19**(6): p. 474-80.
51. Schmidt, K.N., et al., *Validation of the head and neck patient symptom checklist as a nutrition impact symptom assessment tool for head and neck cancer patients.* Supportive Care in Cancer, 2013. **21(1)**: p. 27-34.
52. Chan, M.K.K., et al., *Psychometric validation of a swallowing-related quality-of-life questionnaire: Head and neck cancer survivors' assessment of mealtimes (HN-SAM).* Dysphagia, 2016. **31(2)**: p. 324-325.
53. Hughes, A.P., et al., *HSS Dysphagia and Dysphonia Inventory (HSS-DDI) Following Anterior Cervical Fusion: Patient-Derived, Validated, Condition-Specific Patient-Reported Outcome Measure Outperforms Existing Indices.* The Journal of Bone and Joint Surgery, 2018. **American volume. 100(10)**: p. e66.
54. Hughes, A.P., et al., *Development of an inventory to measure dysphagia and dysphonia after cervical spine surgery.* Spine Journal, 2016. **16(10 Supplement 1)**: p. S355.
55. Heemskerk, A.W., et al., *The Huntington's disease dysphagia scale.* Movement Disorders, 2014. **29(10)**: p. 1312-1316.
56. Pizzorni, N., et al., *The Mealtime Assessment Scale (MAS): Part 1 - Development of a Scale for Meal Assessment.* Folia phoniatrica et logopaedica : official organ of the International Association of Logopedics and Phoniatrics (IALP), 2019: p. 1-13.
57. Hara, K., et al., *Jaw-Opening Force Test to Screen for Dysphagia: Preliminary Results.* Archives of Physical Medicine & Rehabilitation, 2014. **95**(5): p. 867-874.
58. Maeda, K., et al., *Reliability and Validity of a Simplified Comprehensive Assessment Tool for Feeding Support: Kuchi-Kara Taberu Index.* Journal of the American Geriatrics Society, 2016. **64(12)**: p. e248-e252.
59. Govender, R., et al., *Development and preliminary validation of a patient-reported outcome measure for swallowing after total laryngectomy (SOAL questionnaire).* Clinical Otolaryngology, 2012. **37(6)**: p. 452-459.
60. Wang, X.S., et al., *Validation and application of a module of the M. D. Anderson symptom inventory for measuring multiple symptoms in patients with gastrointestinal cancer (the MDASI-GI).* Cancer, 2010. **116(8)**: p. 2053-2063.
61. Sanchez, D., et al., *Psychometric Validation of the M. D. Anderson Symptom Inventory-Head and Neck Module in the Spanish Language.* Journal of Pain and Symptom Management, 2016. **51(6)**: p. 1055-1061.
62. Li, Q., et al., *Chinese version of the Mann assessment of swallowing ability-oral cancer: revision and evaluation of reliability and validity.* Zhonghua kou qiang yi xue za zhi = Zhonghua kouqiang yixue zazhi = Chinese Journal of Somatology, 2021. **56(9)**: p. 914-919.
63. Lim, J.Y., et al., *Use of the maximal phonation test for the screening of dysphagia in stroke patients: a preliminary study.* European journal of physical and rehabilitation medicine, 2020. **56(1)**: p. 41-46.

64. Peters, E.A., et al., *Establishing sensitivity and specificity of the maxwell swallow screen.* Stroke. Conference: American Heart Association/American Stroke Association, 2019. **50**(Supplement 1).
65. McElhiney, J., et al., *The Mayo Dysphagia Questionnaire-30: Documentation of reliability and validity of a tool for interventional trials in adults with esophageal disease.* Dysphagia, 2010. **25(3)**: p. 221-230.
66. Alsubaie, H.M., et al., *Validity and Reliability of an Arabic Version of MD Anderson Dysphagia Inventory (MDADI).* Dysphagia., 2021.
67. Pizzorni, N., et al., *The Mealtime Assessment Scale (MAS): Part 2-Preliminary psychometric analysis. [References]*. 2020: Folia Phoniatrica et Logopaedica:International Journal of Phoniatrics, Speech Therapy and Communication Pathology. Vol.72(3), 2020, pp. 182-193.
68. Rossi, G., et al., *Development and validation of a new mealtime assessment tool (MAT) for patients with dysphagia: A cross sectional study.* Clinica Terapeutica, 2020. **171(4)**: p. E346-E356.
69. Liu, F., et al., *Acceptability of oral solid medicines in older adults with and without dysphagia: A nested pilot validation questionnaire based observational study.* International Journal of Pharmaceutics, 2016. **512(2)**: p. 374-381.
70. Westergren, A.G., et al., *Minimal eating observation form: Reliability and validity.* Journal of Nutrition, Health and Aging, 2009. **13(1)**: p. 6-11.
71. Westergren, A. and D. Melgaard, *The Minimal Eating Observation Form–II Danish Version: Psychometric and Metrological Aspects.* Journal of Nursing Measurement, 2020. **28**(1): p. 168-184.
72. Vallén, C., P. Hagell, and A. Westergren, *Validity and user-friendliness of the minimal eating observation and nutrition form - Version II (MEONF - II) for undernutrition risk screening.* Food and Nutrition Research, 2011. **55**(1): p. 5801.
73. Nishiwaki, K., et al., *Identification of a simple screening tool for dysphagia in patients with stroke using factor analysis of multiple dysphagia variables.* Journal of Rehabilitation Medicine, 2005. **37**(4): p. 247-51.
74. El-Rabbany, M., et al., *Development of the Medication-Related Osteonecrosis of the Jaw Quality of Life Scale (MRONJ-QoL).* Journal of Oral and Maxillofacial Surgery, 2020. **78(10 Supplement)**: p. e28-e29.
75. Bartolome, G., et al., *Validation of the Munich Swallowing Score (MUCSS) in patients with neurogenic dysphagia: A preliminary study.* NeuroRehabilitation, 2021. **49(3)**: p. 445-457.
76. Sakin, Y.S., et al., *Validation and psychometric properties of the Turkish version of Neuromuscular disease Swallowing Status Scale (NdSSS) in patients with oro-pharygo-esopha-geal dysphagia in neuromuscular disorders.* Acta Gastro-Enterologica Belgica, 2022. **85(1)**: p. 21-27.
77. Cummings, J., et al., *Sensitivity and Specificity o f a Nurse Dysphagia Screen in Stroke Patients.* Medical-Surgical Nursing, 2015. **24**(4): p. 219-222.
78. Daniels, S.K., et al., *Rapid Aspiration Screening for Suspected Stroke: Part 1: Development and Validation.* Archives of Physical Medicine and Rehabilitation, 2016. **97(9)**: p. 1440-1448.
79. Campbell, G.B., et al., *Nursing bedside dysphagia screen: Is it valid?* Journal of Neuroscience Nursing, 2016. **48**(2): p. 75-79.
80. Ellis, A.L. and R.R. Hannibal, *Nursing swallow screens: Why is testing water only not enough?* Journal of Neuroscience Nursing, 2013. **45(5)**: p. 244-253.
81. Bravata, D.M., et al., *Comparison of two approaches to screen for dysphagia among acute ischemic stroke patients: nursing admission screening tool versus National Institutes of Health Stroke Scale.* Journal of Rehabilitation Research & Development, 2009. **46**(9): p. 1127-1133.
82. Papadopoulou, S.L., et al., *Adaptation and Assessment of Reliability and Validity of the Greek Version of the Ohkuma Questionnaire for Dysphagia Screening.* International Archives of Otorhinolaryngology, 2017. **21(1)**: p. 58-65.
83. Stiff, P.J., et al., *Reliability and validity of a patient self-administered daily questionnaire to assess impact of oral mucositis (OM) on pain and daily functioning in patients undergoing autologous hematopoietic stem cell transplantation (HSCT).* Bone Marrow Transplantation, 2006. **37(4)**: p. 393-401.
84. Magalhaes Junior, H.V., et al., *Validity Evidence of an Epidemiological Oropharyngeal Dysphagia Screening Questionnaire for Older Adults.* Clinics (Sao Paulo, Brazil), 2020. **75**: p. e1425.

85. Karnell, M.P. and N.M. Rogus, *Comparison of clinician judgments and measurements of swallow response time: A preliminary report.* Journal of Speech, Language, and Hearing Research, 2005. **48(6)**: p. 1269-1279.
86. Sheppard, J.J., et al., *Validation of the choking risk assessment and pneumonia risk assessment for adults with intellectual and developmental disability (IDD).* Dysphagia, 2017. **32(6)**: p. 852.
87. Ross, K.B. and R.T. Wertz, *Discriminative validity of selected measures for differentiating normal from aphasic performance.* American Journal of Speech-Language Pathology, 2003. **12(3)**: p. 312-319.
88. Johnson, K.L., et al., *Validation of a Postextubation Dysphagia Screening Tool for Patients After Prolonged Endotracheal Intubation.* American journal of critical care : an official publication, American Association of Critical-Care Nurses, 2018. **27(2)**: p. 89-96.
89. Gandolfo, C., et al., *The predictive dysphagia score (PreDyScore) in the short- and medium-term post-stroke: a putative tool in PEG indication.* Neurological Sciences, 2019. **40**(8): p. 1619-1626.
90. Kalf, J.G., et al., *Reproducibility and validity of patient-rated assessment of speech, swallowing, and saliva control in parkinson's disease.* Archives of Physical Medicine and Rehabilitation, 2011. **92(7)**: p. 1152-1158.
91. Magalhaes Jr, H.V., et al., *Screening for deglutition disorders in older people (RADI): Validity evidence based on test content and response process.* Dysphagia, 2015. **30(2)**: p. 253.
92. Chepeha, D.B., et al., *Hemiglossectomy tongue reconstruction: Modeling of elevation, protrusion, and functional outcome using receiver operator characteristic curve.* Head and Neck, 2016. **38(7)**: p. 1066-1073.
93. Brogan, L., D. Leavy, and M. Walshe, *Development and validation of the ROC scale in adults with intellectual disability.* Dysphagia, 2017. **32(1)**: p. 135.
94. Broadley, S., et al., *Predicting prolonged dysphagia in acute stroke: the Royal Adelaide Prognostic Index for Dysphagic Stroke (RAPIDS).* Dysphagia, 2005. **20**(4): p. 303-10.
95. Scarponi, L., et al., *Validation into Italian of the royal Brisbane women's hospital dysphagia screening tool.* Dysphagia, 2015. **30(5)**: p. 654-655.
96. Russell-Dalton, A., C. Fennessy, and M.Y. Morgan, *Prospective validation of the royal free hospital gastrostomy risk score.* Gut, 2019. **68(Supplement 2)**: p. A183-A184.
97. Akhtar, U., et al., *Construct Validation of Three Nutrition Questions Using Health and Diet Ratings in Older Canadian Males Living in the Community.* Canadian Journal of Dietetic Practice & Research, 2015. **76**(4): p. 194-199.
98. Edmiaston, J.M., L.T. Connor, and A.L. Ford, *SWALLOW-3D, a simple 2-minute bedside screening test, detects dysphagia in acute stroke patients with high sensitivity when validated against video-fluoroscopy.* Stroke, 2011. **42(3)**: p. e352.
99. Kagaya, H., et al., *Simple swallowing provocation test has limited applicability as a screening tool for detecting aspiration, silent aspiration, or penetration.* Dysphagia, 2010. **25(1)**: p. 6-10.
100. Jiang, J.L., et al., *Evaluation of the Chinese version of the swallowing screen in stroke patients with dysphagia.* Tzu Chi Medical Journal, 2019. **31(4)**: p. 270-275.
101. Lindroos, E. and K. Johansson, *Free from Dysphagia? A Test Battery to Differentiate Between Mild and No Dysphagia.* Dysphagia, 2022. **37**(3): p. 501-509.
102. Crary, M.A., G.D. Carnaby, and I. Sia, *Spontaneous swallow frequency compared with clinical screening in the identification of dysphagia in acute stroke.* Journal of Stroke and Cerebrovascular Diseases, 2014. **23(8)**: p. 2047-2053.
103. Branco, L.L., et al., *The Swallowing Clinical Assessment Score in Parkinson's Disease (SCAS-PD) Is a Valid and Low-Cost Tool for Evaluation of Dysphagia: A Gold-Standard Comparison Study.* Journal of Aging Research, 2019. **2019**: p. 2019.
104. Cardoso, A.R., et al., *Cross-cultural adaptation and validation of the Swallowing Disturbance Questionnaire and the Sialorrhea Clinical Scale in Portuguese patients with Parkinson's disease.* Logopedics Phoniatrics Vocology, 2021. **46**(4): p. 163-170.
105. Govender, R., et al., *Psychometric evaluation of the Swallowing Outcomes After Laryngectomy (SOAL) patient-reported outcome measure.* Head Neck, 2016. **38 Suppl 1**: p. E1639-45.
106. Khaldoun, E., V. Woisard, and E. Verin, *Validation in French of the SWAL-QOL scale in patients with oropharyngeal dysphagia.* Gastroenterologie Clinique et Biologique, 2009. **33(3)**: p. 167-171.
107. Albinsson, S., et al., *Validation of the Swedish Watson Dysphagia Scale for adult patients with eosinophilic esophagitis.* Diseases of the Esophagus, 2022. **22**.

108. Liang, G., et al., *Comparison of Bazaz scale, Dysphagia Short Questionnaire, and Hospital for Special Surgery-Dysphagia and Dysphonia Inventory for Assessing Dysphagia Symptoms After Anterior Cervical Spine Surgery in Chinese Population.* Dysphagia., 2021.
109. Vogel, A.P., et al., *Clinical assessment of dysphagia in neurodegeneration (CADN): development, validity and reliability of a bedside tool for dysphagia assessment.* Journal of Neurology, 2017. **264(6)**: p. 1107-1117.
110. Kunieda, K., et al., *Reliability and validity of a tool to measure the severity of dysphagia: The food intake LEVEL scale.* Journal of Pain and Symptom Management, 2013. **46(2)**: p. 201-206.
111. De Bellis, F., et al., *Assessing communicative abilities in patients with severe acquired brain injury by Functional Communication Measures.* European Journal of Neurology, 2021. **28 (SUPPL 1)**: p. 85.
112. Piot, I., et al., *The Progressive Supranuclear Palsy Clinical Deficits Scale.* Movement Disorders, 2020. **35(4)**: p. 650-661.
113. Vitali, C., et al., *Oropharyngolaryngeal disorders in scleroderma: Development and validation of the SLS scale.* Dysphagia, 2010. **25(2)**: p. 127-138.
114. Simon, S.R., et al., *Malnutrition screening in head and neck cancer patients with oropharyngeal dysphagia.* Clinical Nutrition ESPEN, 2021. **44**: p. 348-355.
115. Timmeren, D.E.A., et al., *The convergent validity of a Dutch Screening tool for Dysphagia (Signaleringslijst Verslikken) for people with severe or profound intellectual and multiple disabilities.* Journal of Applied Research in Intellectual Disabilities, 2019. **32**(4): p. 994-1001.
116. Osawa, A., S. Maeshima, and N. Tanahashi, *Water-swallowing test: Screening for aspiration in stroke patients.* Cerebrovascular Diseases, 2013. **35(3)**: p. 276-281.
117. Persson, J., et al., *Validation of instruments for the assessment of dysphagia due to malignancy of the esophagus.* Diseases of the Esophagus, 2019. **32**(9): p. doy 125.
118. Nakamori, M., et al., *Prediction of Pneumonia in Acute Stroke Patients Using Tongue Pressure Measurements.* PLoS One, 2016. **11**(11): p. e0165837.
119. Martino, R., E. Maki, and N. Diamant, *Identification of dysphagia using the Toronto Bedside Swallowing Screening Test (TOR-BSST©): are 10 teaspoons of water necessary?* International Journal of Speech-Language Pathology, 2014. **16**(3): p. 193-198.
120. Koiwai, K., et al., *Validation of the total dysphagia risk score (TDRS) as a predictive measure for acute swallowing dysfunction induced by chemoradiotherapy for head and neck cancers.* Radiotherapy and Oncology, 2010. **97(1)**: p. 132-135.
121. Momosaki, R., et al., *Applicability of the two-step thickened water test in patients with poststroke Dysphagia: a novel assessment tool for paste food aspiration.* Journal of Stroke & Cerebrovascular Diseases, 2013. **22**(6): p. 817-821.
122. Radhakrishnan, S., et al., *Usefulness of a modified questionnaire as a screening tool for swallowing disorders in Parkinson disease: A pilot study.* Neurology India, 2019. **67**(1): p. 118-122.
123. Murphy, B.A., et al., *Reliability and validity of the Vanderbilt Head and Neck Symptom Survey: A tool to assess symptom burden in patients treated with chemoradiation.* Head and Neck, 2010. **32(1)**: p. 26-37.
124. Rinkel, R.N.P.M., et al., *Patient-reported symptom questionnaires in laryngeal cancer: Voice, speech and swallowing.* Oral Oncology, 2014. **50(8)**: p. 759-764.
125. Guillén-Solà, A., et al., *Usefulness of the volume-viscosity swallow test for screening dysphagia in subacute stroke patients in rehabilitation income.* NeuroRehabilitation, 2013. **33**(4): p. 631-638.
