# Supplementary material for: Reliability and Validity of Non-Instrumental Clinical Assessments for Adults with Oropharyngeal Dysphagia: A Systematic Review
Source: J Clin Med. 2023 Jan 16;12(2):721. doi: 10.3390/jcm12020721 (PMC9861493; doi:10.3390/jcm12020721)
Supplement: Supplementary file 1 [file jcm-12-00721-s001.zip › Supplementary FileS5 Study Quality QualSyst.pdf]

Supplementary File S5. Reliability and Validity of Non-instrumental Clinical Assessments for Adults with Oropharyngeal Dysphagia: A systematic review.

Methodological quality of included studies based on QualSyst critical appraisal tool by Kmet et al. 2004 [1]

QualSyst Item / Reference	Question/objective sufficiently described	Study design evident and appropriate?	Method of subject/ comparison group selection or source of information/ input variables described	Subject and comparison group, if applicable characteristics sufficiently described?	Outcome and if applicable exposure measures well defined and robust to measurement/	Sample size appropriate?	Analytic methods described/justified and appropriate	Some estimate of variance is reported for the main results	Results reported in sufficient detail?	Conclusions supported by the results?	Methodological Quality [1]
Antonios et al. 2010 [2]	2	2	2	2	2	2	2	2	2	2	Strong 100%
Borders et al. 2021 [3]	2	2	2	2	2	1	2	2	2	2	Strong 95%
Carnaby & Crary 2014 [4]	2	2	2	2	2	2	2	2	2	2	Strong 100%
Chun et al. 2017 [5]	2	2	2	2	2	2	1	2	2	2	Strong 95%
Crary et al. 2005 [6]	2	2	2	2	2	2	1	2	2	2	Strong 95%
Dungan et al. 2019 [7]	2	2	2	2	2	2	2	1	2	2	Strong 95%
Everton et al. 2020 [8]	2	2	2	1	2	2	1	2	2	2	Strong 90%
Francis-Bacz et al. 2013 [9]	2	2	2	2	2	2	2	2	2	2	Strong 100%
González-Fernández et al. 2011 [10]	2	2	2	2	2	2	2	2	2	2	Strong 100%
Huckabee et al. 2018 [11]	2	2	2	2	2	2	2	2	2	2	Strong 100%
Hyun et al. 2021 [12]	2	2	2	2	2	2	1	2	2	2	Strong 95%
Ishii et al. 2021 [13]	2	2	2	2	2	2	0	2	2	2	Strong 90%
Ji et al. 2019 [14]	2	2	2	2	2	2	1	2	2	2	Strong 95%
Karsten et al. 2021 [15]	2	2	2	2	2	2	2	2	2	2	Strong 100%

QualSyst Item / Reference	Question/objective sufficiently described	Study design evident and appropriate?	Method of subject/ comparison group selection or source of information/ input variables described	Subject and comparison group, if applicable characteristics sufficiently described?	Outcome and if applicable exposure measures well defined and robust to measurement/	Sample size appropriate?	Analytic methods described/justified and appropriate	Some estimate of variance is reported for the main results	Results reported in sufficient detail?	Conclusions supported by the results?	Methodological Quality [1]
Kothari et al. 2021 [16]	2	2	2	2	2	2	2	2	2	2	Strong 100%
Kwon et al. 2019 [17]	2	2	2	2	2	2	2	2	2	2	Strong 100%
Lambert et al. 2003 [18]	2	2	2	2	2	2	2	2	2	2	Strong 90%
Lambert et al. 2005 [19]	2	2	2	2	2	2	0	2	2	2	Strong 90%
Lambert et al. 2006 [20]	2	2	2	2	2	2	1	2	2	2	Strong 95%
McMicken & Muzzy 2009a [21]	2	2	2	2	2	2	2	2	2	2	Strong 100%
McMicken & Muzzy 2009b [22]	2	2	2	2	2	2	1	2	2	2	Strong 95%
McMicken et al. 2010 [23]	2	2	2	2	2	2	2	2	2	2	Strong 100%
Mitani et al. 2018 [24]	2	2	2	2	2	2	2	2	2	2	Strong 100%
Nordio et al. 2018 [25]	2	2	2	2	2	2	1	1	2	2	Strong 90%
Oliveira et al. 2015 [26]	2	2	2	2	2	2	2	2	2	2	Strong 100%
Oliveira-Kumakura et al. 2018 [27]	2	2	2	2	2	2	2	2	2	2	Strong 100%
Oliveira-Kumakura et al. 2019 [28]	2	2	2	2	2	2	2	2	2	2	Strong 100%
Sellers et al. 2013 [29]	2	2	2	2	2	2	2	2	2	2	Strong 100%
Sheppard et al. 2014 [30]	2	2	2	2	2	2	2	2	2	2	Strong 100%
Steele et al. 2018 [31]	2	2	1	2	2	2	2	2	2	2	Strong 95%
Todaro et al. 2021 [32]	2	2	2	2	2	2	2	2	2	2	Strong 100%
Uhm et al. 2019 [33]	2	2	2	2	2	2	1	2	2	2	Strong 95%

[1] Methodological quality: strong > 80%; good 60–79%; adequate 50–59%; poor < 50%

References

1. Kmet, L.M., L.S. Cook, and R.C. Lee, *Standard quality assessment criteria for evaluating primary research papers from a variety of fields.* 2004.
2. Antonios, N., et al., *Analysis of a Physician Tool for Evaluating Dysphagia on an Inpatient Stroke Unit: The Modified Mann Assessment of Swallowing Ability.* Journal of Stroke and Cerebrovascular Diseases, 2010. **19(1)**: p. 49-57.
3. Borders, J.C., et al., *Objective and Subjective Clinical Swallowing Outcomes via Telehealth: Reliability in Outpatient Clinical Practice.* American Journal of Speech-Language Pathology, 2021. **30**: p. 598-608.
4. Carnaby, G.D. and M.A. Crary, *Development and validation of a cancer-specific swallowing assessment tool: MASA-C.* Supportive Care in Cancer, 2014. **22(3)**: p. 595-602.
5. Chun, M.H., D. Kim, and M.C. Chang, *Comparison of dysphagia outcomes between rostral and caudal lateral medullary infarct patients.* International Journal of Neuroscience, 2017. **127**(11): p. 965-970.
6. Crary, M.A., G.D. Carnaby Mann, and M.E. Groher, *Initial psychometric assessment of a functional oral intake scale for dysphagia in stroke patients.* Archives of Physical Medicine and Rehabilitation, 2005. **86(8)**: p. 1516-1520.
7. Dungan, S., et al., *Comparative Validity of the American Speech-Language-Hearing Association's National Outcomes Measurement System, Functional Oral Intake Scale, and G-Codes to Mann Assessment of Swallowing Ability Scores for Dysphagia.* American Journal of Speech-Language Pathology, 2019. **28(2)**: p. 424-429.
8. Everton, L.F., et al., *Psychometric assessment and validation of the dysphagia severity rating scale in stroke patients.* Scientific Reports, 2020. **10(1)**: p. 1-10.
9. Francis-Bacz, C., S. Wood-Dauphinee, and E. Gisel, *The Discriminative Validity of the McGill Ingestive Skills Assessment.* Physical & Occupational Therapy In Geriatrics, 2013. **31**(2): p. 148-158.
10. González-Fernández, M., M.T. Sein, and J.B. Palmer, *Clinical experience using the Mann assessment of swallowing ability for identification of patients at risk for aspiration in a mixed-disease population.* American Journal of Speech-Language Pathology, 2011. **20**(4): p. 331-336.
11. Huckabee, M.L., O. Apperley, and C. Lazarus, *Quantitative measurement of solid bolus ingestion in post-radiation head and neck cancer patients.* Dysphagia, 2018. **33(4)**: p. 530-531.
12. Hyun, S.E., Y.G. Yi, and H.-I. Shin, *Reliability and Validity of the Eating and Drinking Ability Classification System in Adults with Cerebral Palsy.* Dysphagia, 2021. **36**(3): p. 351-361.
13. Ishii, M., et al., *Higher Activity and Quality of Life Correlates with Swallowing Function in Older Adults with Low Activities of Daily Living.* Gerontology, 2021: p. 1-9.
14. Ji, E.K., et al., *Is the modified Mann Assessment of Swallowing Ability useful for assessing dysphagia in patients with mild to moderate dementia?* Journal of Clinical Neuroscience, 2019. **70**: p. 169-172.
15. Karsten, R.T., et al., *The Timed Swallowing Proficiency for Eating and Drinking (SPEAD) Test: Development and Initial Validation of an Instrument to Objectify (Impaired) Swallowing Capacity in Head and Neck Cancer Patients.* Dysphagia, 2021. **36**(6): p. 1072-1087.
16. Kothari, S., et al., *The Test of Masticating and Swallowing Solids (TOMASS): Reliability, Validity and Normative Data for the Adult Indian Population.* Indian Journal of Otolaryngoly and Head & Neck Surgery, 2021. **73**(3): p. 327-332.
17. Kwon, S., et al., *Assessment of Aspiration Risk Using the Mann Assessment of Swallowing Ability in Brain-Injured Patients With Cognitive Impairment.* Frontiers in Neurology, 2019. **10**: p. 1264.
18. Lambert, H.C., et al., *McGill Ingestive Skills Assessment (MISA): Development and first field test of an evaluation of functional ingestive skills of elderly persons.* Dysphagia, 2003. **18(2)**: p. 101-113.
19. Lambert, H.C., et al., *The McGill ingestive skills assessment predicts time to death in an elderly population with neurogenic dysphagia: preliminary evidence.* Dysphagia, 2005. **20**(2): p. 123-32.
20. Lambert, H.C., et al., *Psychometric Testing of the McGill Ingestive Skills Assessment.* American Journal of Occupational Therapy, 2006. **60**(4): p. 409-419.
21. McMicken, B.L. and C.L. Muzzy, *Functional outcomes of standard dysphagia treatment in first time documented stroke patients.* Disability and Rehabilitation, 2009. **31(10)**: p. 806-817.

22. McMicken, B.L. and C.L. Muzzy, *Prognostic indicators of functional outcomes in first time documented acute stroke patients following standard dysphagia treatment.* Disability and Rehabilitation, 2009. **31**(26): p. 2196-2203.
23. McMicken, B.L., C.L. Muzzy, and S. Calahan, *Retrospective ratings of 100 first time-documented stroke patients on the Functional Oral Intake Scale.* Disability and Rehabilitation, 2010. **32(14)**: p. 1163-1172.
24. Mitani, Y., et al., *Relationship between the Functional Independence Measure and Mann Assessment of Swallowing Ability in hospitalized patients with pneumonia.* Geriatrics & Gerontology International, 2018. **18**(12): p. 1620-1624.
25. Nordio, S., et al., *The correlation between pharyngeal residue, penetration/aspiration and nutritional modality: a cross-sectional study in patients with neurogenic dysphagia.* Acta Otorhinolaryngologica Italica, 2020. **40**(1): p. 38-43.
26. Oliveira, A.R.d.S.-., et al., *Construction and validation of indicators and respective definitions for the nursing outcome Swallowing Status.* Revista Latino-Americana de Enfermagem, 2015. **23**(3): p. 450-457.
27. Oliveira-Kumakura, A.R.S., et al., *Clinical Validation of the Nursing Outcome "Swallowing Status" in People with Stroke: Analysis According to the Classical and Item Response Theories.* International Journal of Nursing Knowledge, 2018. **29**(4): p. 234-241.
28. Oliveira-Kumakura, A.R.S., J.B. Alonso, and E.C. de Carvalho, *Psychometric Assessment of the Nursing Outcome Swallowing Status: Rasch Model Approach.* International Journal of Nursing Knowledge, 2019. **30(4)**: p. 197-202.
29. Sellers, D., et al., *Development of an Eating and Drinking Ability Classification System (EDACS) for people with cerebral palsy.* Developmental Medicine and Child Neurology, 2013. **2)**: p. 49-50.
30. Sheppard, J.J., R. Hochman, and C. Baer, *The Dysphagia Disorder Survey: Validation of an assessment for swallowing and feeding function in developmental disability.* Research in Developmental Disabilities, 2014. **35**(5): p. 929-942.
31. Steele, C.M., et al., *Creation and Initial Validation of the International Dysphagia Diet Standardisation Initiative Functional Diet Scale.* Archives of Physical Medicine & Rehabilitation, 2018. **99**(5): p. 934-944.
32. Todaro, F., et al., *The test of masticating and swallowing solids (tomass): Reliability and validity in patients with dysphagia.* International Journal of Language & Communication Disorders, 2021. **56**(3): p. 558-566.
33. Uhm, K.E., et al., *The Easy Dysphagia Symptom Questionnaire (EDSQ): a new dysphagia screening questionnaire for the older adults.* European Geriatric Medicine, 2019. **10(1)**: p. 47-52.
